# Development of a digital mental health intervention for youth with ADHD: exploring youth perspectives on wants, needs, and barriers

**DOI:** 10.3389/fdgth.2024.1386892

**Published:** 2024-07-10

**Authors:** Maren Helene Rinke Storetvedt, Smiti Kahlon, Karin Berg, Ingeborg Alvheim Sundfjord, Tine Nordgreen

**Affiliations:** ^1^ Research Centre for Digital Mental Health Services, Division of Psychiatry, Haukeland University Hospital, Bergen, Norway; ^2^ Department of Global Public Health and Primary Care, Faculty of Medicine, University of Bergen, Bergen, Norway; ^3^ Department of Clinical Psychology, Faculty of Psychology, University of Bergen, Bergen, Norway

**Keywords:** attention deficit hyperactivity disorder, adolescents, digital intervention, person-based approach, user-centered, engagement, qualitative

## Abstract

**Background:**

Attention deficit hyperactivity disorder (ADHD) is a mental health disorder affecting five to eight percent of children and youth worldwide. Inattention, impulsivity, and hyperactivity are core symptoms, which often leads to comorbid disorders and impairments. Increased prevalence of ADHD among youth requires development of accessible and scalable interventions. Digital interventions for ADHD show promising results for adults, yet insight into youths perspectives and needs from digital ADHD interventions is lacking. This study is set in a person-based approach and explore what youths want and need from a therapist-guided digital intervention for ADHD.

**Methods:**

Exploratory individual interviews were conducted with youths aged 13–16 years diagnosed with ADHD (*N *= 16). Participants with an ADHD diagnosis were recruited primarily via social media. The interview guide was based on research, treatment guidelines, and clinical expertise. The study applied reflexive thematic analysis, within a Big Q framework. Codes and themes were generated in Nvivo.

**Results:**

Four main themes and sub-themes were generated: (1) Tailoring the intervention to youths with ADHD (Push the right buttons; Stumbling blocks), (2) Managing ADHD (Planning and Focus; Regulation and Balance; Social interactions), (3) Me and my ADHD (Insight and Understanding; Accept and Normalization), and (4) Balance between support and independence.

**Discussion:**

The findings suggest that youths with ADHD prefer stimulating and interactive treatment and are aversive to overwhelming, passive content. The intervention and therapist should encourage empowerment by supporting the youths autonomy in managing their ADHD. Future research is needed to investigate feasibility for person-based approaches to digital mental health treatments. Furthermore, parent perspectives on digital treatment for youths with ADHD should be investigated.

## 1 Introduction

Attention deficit hyperactivity disorder (ADHD) is a mental health disorder that affects five to eight percent of children and young people worldwide (1). In recent years, an increase in ADHD diagnoses among children and youths have been reported (2). ADHD presents a costly societal challenge and can lead to severe difficulties for children, youth and their families (3). Inattention, impulsivity, and hyperactivity are core symptoms of the disorder (1). The diagnosis is set if the symptoms manifest in childhood, are prevalent over time and constitute at least a moderate psychological, social, school and/or work impairment (4). In addition, many with ADHD experience functional challenges including self-organization, goal-directed actions, self-regulation, inhibition, working memory, and emotion regulation (5).

The National Institute for Health and Care Excellence (4) recommend a comprehensive ADHD treatment that covers behavioral, psychological, educational, or occupational needs, consisting of pharmacological and non-pharmacological treatment. Reviews on pharmacological controlled trials report medium (*d *= 0.64) (6) and large (7) effect sizes on core symptoms of ADHD in youths. However, youths demonstrate low adherence to pharmacological treatment and many experience side effects (8–10).

Non-pharmacological interventions have been shown to have positive effects on ADHD in many studies (11, 12). Several studies have documented Cognitive behavioral therapy (CBT) as effective (13–15), and the treatment is recommended for ADHD in both adults and youths (4). Internet delivered CBT is considered a beneficial format, as few patients with ADHD are offered CBT treatment and there is a limited access to CBT therapists (16), with even fewer CBT therapists specializing in ADHD. A recent randomized controlled trial on adults with ADHD found that a digital self-guided intervention based on CBT promoted increased quality of life and reduced ADHD symptoms (17).

However, there is a lack of studies on non-pharmacological treatments outside the adult and preschool age-group (18). This is important to note as youth ADHD manifests differently compared to other age-groups (18). The transition from child to youth leads to a reduction in core symptoms of ADHD such as inattention and hyperactivity, yet youths with ADHD demonstrate an increase in general psychopathology (19). Moreover, research indicates that non-pharmacological interventions for youth with ADHD have little impact on aspects such as peer functioning (20). This is of relevance as social relations becomes increasingly important and are affected by core difficulties related to self-regulation (18). Both children and youths have above-average parenting needs (4), yet parenting youth with ADHD also requires a balance between support and autonomy (21). Furthermore, compared to adults, youths demonstrate lower adherence to both pharmacological and non-pharmacological treatment, indicating a need to promote treatment engagement in youths with ADHD (22). It is therefore essential to develop appropriate treatments for this user-group.

Although insight into youth perspectives on unmet needs can reduce non-compliance and drop-out from treatment, there is little research on the topic (23). The person-based approach is recommended for providing tailored diagnosis management to youths with ADHD and for developing successful and engaging digital health interventions (4, 24, 25). In order to understand the youth perspectives and thereby facilitate treatment engagement, we will conduct a qualitative explorative study in accordance with a person-based approach to digital intervention development (25).

The aim of this study is to gain knowledge about what the participants find useful and necessary in a novel digital health intervention and potential barriers. This study will contribute to knowledge-development within the overall intervention development framework, the person-based approach (25). The research questions are:

1. What do youths between 13 and 16 years diagnosed with ADHD want and need from a novel digital intervention?
2. What are the barriers related to a novel digital intervention for youths with ADHD?

## 2 Materials and methods

### 2.1 Ethical considerations

The study was approved by the Regional Committee for Medical Research Ethics of Western Norway (520625).

### 2.2 Design

Exploratory individual semi-structured interviews.

### 2.3 Participants and recruitment

**Figure 1:**
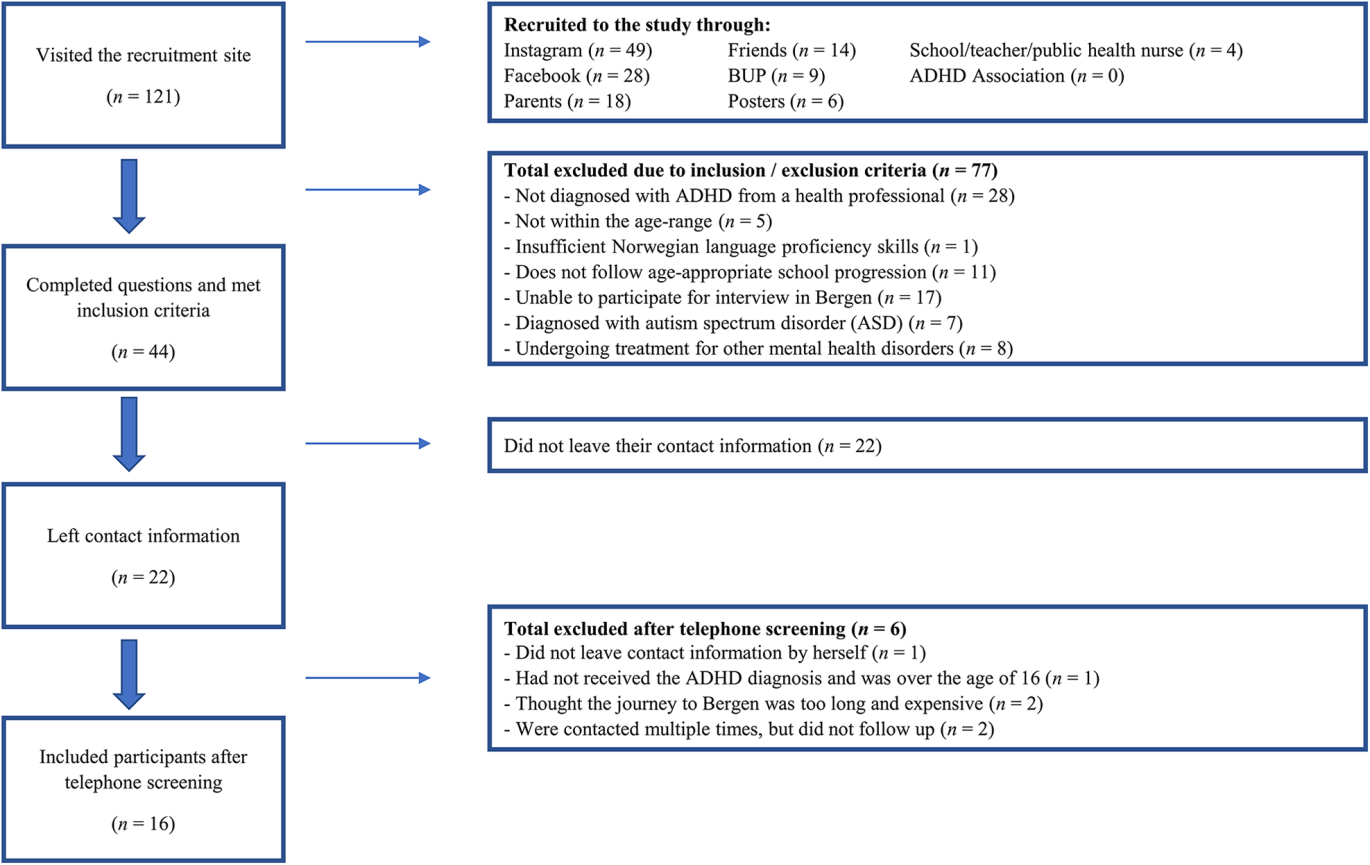
Participants included and excluded.

The participants consisted of a convenience sample of youths aged 13–16 years diagnosed with ADHD (*N *= 16). In Norway clinicians are currently using DSM-5 for diagnosing ADHD and code after ICD-10 (26). The recruitment was conducted between the 10th of October 2022 to the 2nd of January 2023. Minimal sample size was set to 12 participants, as this has been found to be adequate to provide saturation (27). The recruitment catchment area was set in Bergen and surrounding area. We primarily recruited participants via the social media channels Instagram and Facebook with a digital poster targeting youths in the catchment area. We shared the digital poster and distributed physical posters to relevant organizations and institutions (see Figure 1 for more details about the recruitment). By scanning a QR-code the youths were routed to a recruitment-site. The site contained information about the study and screening questions that evaluated if the study was eligible. The screening questions were based on the following inclusion and exclusion criteria:

Inclusion criteria: (a) between 13 and 16 years; (b) ADHD diagnosis; (c) can operate and access PC/mobile phone/tablet; (d) Norwegian language proficiency; (e) face-to-face participation on the interviews. Exclusion criteria: (a) Autism spectrum disorder; (b) in immediate need or undergoing treatment for other mental health disorders; (c) does not follow a regular school plan.”

The criterion “Does not follow a regular school plan” was set as an exclusion criterion to operationalize that the study might be too burdensome for some youths with ADHD.

The first author contacted participants or their parents via telephone to confirm their interest in participating in the study, ensure that the study was a good fit, and invited them to interviews at Haukeland University Hospital. For participants younger than 16 years consent was obtained from both parents. Participants aged 16 years gave their own consent to participate in the study.

The participants were compensated for their time with a gift card worth 300 NOK. We acknowledge that this might have motivated some to participate in the study, yet we consider this a reasonable compensation for the youths’ contribution and time.

### 2.4 Semi-structured interviews

Semi-structured interviews were conducted between 23rd of October 2022 and 11th of January 2023. None of the included participants dropped out of the study. The youths were given the choice to have a parent present, where six interviews were conducted with parents present. Ten interviews were conducted by PhD candidate and the first author (M.S; female) while six interviews were conducted by two female students in clinical psychology (three interviews each); K.B., I.A.S. M.S. has previous experience with interviews and provided guidance and training for the co-authors. Only one interviewer was present during each interview, to create a less formal atmosphere.

The first author (M.S.) had the main responsibility for the interview process. With a background from health promotion she was inspired by a salutogenic perspective when, in collaboration with the last author, she developed the interview guide. In contrast and supplement to pathogenesis, salutogenesis focuses on what makes us healthy as opposed to what makes us ill (28). An example of how this was conveyed to the youths is that we referred to the intervention as an intervention to master ADHD, rather than an intervention to treat ADHD.

The interview guide was informed by research, guidelines for ADHD treatment, and clinical expertise, which made us aware of a need to address social functioning and emotions. We wanted to explore these aspects as well as covering generally relevant areas related to ADHD and let the youths elaborate on topics they considered especially important (for further details see Supplementary Interview Guide). The guide was piloted with a 16-year-old youth (not diagnosed with ADHD) to identify potentially difficult language and other adjustment needs. We adjusted the interview guide by incorporating some pictures and varied formats prevent the participants from losing attention due to monotonous questioning.

Before the interview started, participants were informed about the aim of the study, the interviewers’ role, how the information from the interviews would be used and their rights. An audio recorder was used, and the interviewer made notes during the interviews. Transcriptions were performed manually in word, and transcription rules and written form was agreed upon. Interview files were transcribed by the interviewers and an assistant.

The interviews varied in length, with a mean duration of 44.56 min (SD = 8.74). The shortest interview lasted 33 min, while the longest lasted 65 min. All interviews were conducted on-site without any disruptions.

### 2.5 Reflexivity—analytic approach

This study is set in a Big Q framework which is a qualitative paradigm that constitutes the basis of our chosen approach—reflexive thematic analysis by Braun and Clarke (29, 30). The Big Q framework rejects small q/postpositivist ideals and tools like interrater reliability, avoiding researcher bias, coding frameworks and instead promote subjectivity, reflective writing, contextualized and situated knowledge (30).

The reflexive thematic approach imply that our subjectivity as researchers is a research tool that has affected the analytic process. Reflective writing was applied throughout the research process (30) and was helpful to address factors that have shaped the interview process such as: unbalanced power dynamics, having parents present during interviews, interview questions, setting and more. Some parents occasionally answered on behalf of their children or helped them remember or stay focused during the interview. As this study focus on the youths’ perspectives, we did not include parents’ answers in the analysis. However, the presence of parents likely influenced the responses given in the interviews either by promoting comfort to share or by causing some youths to withhold information. The analytic approach was set in a relativist-constructionist framework, promoting that we cannot extract objective reality from the data, as reality is a construct of societal and individual sense making. Further we used an inductive analytic approach that allowed for a bottom-up analysis and focused on semantic meaning (30).

The majority of the authors are working in a research centre devoted to researching and developing digital mental health services. We have reflected on how this could make us prone to focus on the positive sides of digital treatment, and less critical of the pitfalls of the digital solutions. This have shaped the whole research process from the questions we asked to the interpretations we made and the way we framed the discussion. This study is rooted in a person-based approach (25), and we have promoted a resource-oriented perspective on the participants and their perspectives throughout the study.

### 2.6 Data analysis

Data management and analysis were conducted in Nvivo (2020R1). The six steps of reflexive thematic analysis (29, 30) were applied: familiarization, coding, generating themes, reviewing themes, defining themes, and summarization. Data familiarization started with transposing the audio-files of the interviews. M.S. transposed eight of the ten interviews she conducted, and a research assistant transposed the remaining two. K.B. and I.A.S. transposed their six interviews. Thereafter all sixteen transcripts were read and reread in different order by all three interviewers, notes were made, and impressions discussed. Preliminary coding of six interviews was performed by K.B. and I.A.S. In addition, M.S. coded three interviews and initial impressions of these interviews were discussed. In order to facilitate coherence M.S. continued by coding all sixteen interviews and performed the rest of the analysis alone. Reflexive thematic approach is applicable in team research and is considered particularly suitable for a single researcher (30). After all interviews were coded, initial themes were generated. In the next step transcripts were revisited, and themes were re-evaluated and adjusted. Thematic analysis is not a linear process (30) and codes were merged, and themes changed back and forth in later stages of analysis. Themes and codes were discussed with T.N. in later stages of analysis. The last author did not perform independent coding, but shared valuable perspectives regarding meaning, content and wording that improved the analysis. All co-authors approved the final themes. Lastly, a summary report of main findings was written, and representative quotes chosen for presentation.

## 3 Results

### 3.1 Participants characteristics

The recruitment site had 121 visits and a total number of (*N* = 16) participants were found eligible for exploratory interviews (Figure 1), with 56% females (*n* = 9) and 44% males (*n* = 7). The participants were between 13 and 16 years, with a mean age of 14.75 years (*SD* = 1.18). Nine participants reported that they used medication. None of the participants had an immigrant background. No distinctive differences were observed between the genders, but the interviewers concurred that the age difference was noticeable in how they reflected and the number of inputs they shared. It was evident that the older youths considered substance abuse to be a more relevant topic to address than the younger participants. None of the youths had tried digital treatment for ADHD, and only one youth had received a course related to ADHD management. A digital intervention was considered to be an accessible and suitable format for young people with ADHD. Many reported that their primary source of information about ADHD was the social media channel TikTok. See Table 1 for an overview of the four themes.

**Table 1:** Summary of themes, sub-themes and quotes from exploratory interviews.

Themes	Sub-themes	Example quotes
1: Tailoring the intervention to youths with ADHD: Aspects considered to promote or hinder use	Push the right buttons: Appealing formats, motivating and relevant content	“You don't always want to do things, but if it's fun as well, I think people will use it” (T10). “If it's an app, it should be understandable and give a clear overview, so it isn't frustrating to use” (T14). «Seeing that you improve yourself and get to a new level is a good motivation” (T5).
	Stumbling blocks: Demotivating design and traits to beware of	“Don't press in questions on a long page but have them come one by one after each other” (T9). “(…) when I was diagnosed, I got pamphlets about ADHD, about girls and ADHD, and that would probably be alright for me to read but I will never in the world read a twenty-page long pamphlet. It's okay for parents if they don't also have ADHD… But things that´s been shortened down would be nice. That information is suitable for the person that needs it” (T13). “Don't make text bubbles about like “how to behave” because nobody will find that useful” (T13).
2: Managing ADHD: Wants and needs from the intervention related to everyday struggles	Planning and focus: Mastering organizing and concentration	“That it helps you plan and set up time. For instance if I have homework, how much time off can I take, how many breaks can I take” (T15). “Maybe an exercise or that you find ways of staying more focused. Trick your brain to be able to do it, or to study” (T11). “It would be nice with concentration exercises because that´s the biggest struggle for me with ADHD, or what bothers me the most in everyday life. The fact that I have a lot of energy doesńt bother me, but concentration affects me and my school work a lot” (T14).
	Regulation and balance: Learning to stabilize and take charge of internal life	«It´s like with ADHD you don't think when you do something, you just do it. Don't think about what consequences it might have, how it can be for others, almost like Asperger (…)” (T11). “Finding a pattern you can follow when you get mad, like if you get mad you count to twenty” (T10). «Physical activity, I've heard that it helps for people with ADHD to get the energy out and concentrate the energy in one place” (T12).
	Social interactions: Applying relational skills	“It can be a bit difficult to listen to what others say and not just listen to your own opinion” (T12). “I was very rough when I was a child. So, if I had known how to like, get it out without taking it out on others, that might have been alright” (T13). “Before, I felt that I was far behind socially” (T15).
3: Me and my ADHD: The need for information to understand, and to embrace similarities and individual differences	Insight and understanding: Knowledge is one key to master ADHD	“It is very important to get to know your own ADHD, and what works” (T14). «Very important with information, not just to help people with ADHD, but to help those without ADHD to understand what it's like for folks with ADHD” (T3). “It would be smart for people to get more knowledge about it (medication) because I think it's great, but a lot of people probably think medications for something mental sounds a bit scary” (T6). “(…) if I don't understand why I need to learn something and don't see the point in it, because nobody told what I can use the information for in the future, I quickly get like anti” (T3).
	Accept and Normalization: Wanting belonging, respect and appreciation	“(…) it's nice to see that there's a person that thinks the same as me, feels the same, and then you know you're not completely alone in thinking that” (T16). “If I get a bit irritated or angry they always ask like: “Did you remember to take your medication or have you taken your medication today?” It's very annoying when people ask that, because people who don't have ADHD can also get mad and irritated” (T1). “ADHD is not a dumb thing. It's quite nice to have it because you have a lot of energy” (T10).
4: Balance between support and independence: The delicate balance between providing facilitation and autonomy		“It's good that it's easier to contact someone who has professional knowledge” (T14). “It could be open for parents to be added (in the program). But not like a hawk thing, like: “have you taken your medication?” That's too much, you are supposed to handle it yourself. Maybe you can choose what they get access to” (T13). “It's a bit more fun when you've been involved in deciding what to do” (T2). “Listen to the youth, don't always listen to the adults” (T10).

### 3.2 Theme 1—Tailoring the intervention to youths with ADHD

The core of this theme is that the intervention must be adapted to the prerequisites of youths with ADHD. The theme captures aspects regarding design, functions, content and also ADHD related tendencies that might promote or inhibit the youths from using the intervention. Theme one consists of two sub-themes: Push the right buttons and Stumbling blocks.

#### 3.2.1 Push the right buttons

This sub-theme addresses motivational aspects that the youths believe will encourage usage. Overall, the youths expressed that they wanted the intervention to balance fun and usefulness. Games and Virtual Reality (VR) was considered fun and a good way to socialize. An important motivational factor promoted by seven participants was visible success and progression, for instance by receiving points or reaching new levels.

Half of the participants believed it would be easier to communicate by chat. One teen reflected that: “*Many find it difficult having to show up or talking in real life. On the internet a lot of people are much more open. So it (chat) might be a good thing for those who find it difficult to share things with people (…)”* (*T06*).

Simplicity and overview was stated as a condition for willingness to use the intervention. This was considered important to avoid provoking frustration and overwhelm. Lastly it was pointed out that the intervention must be suitable for youth with ADHD.

#### 3.2.2 Stumbling blocks

The sub-theme covers barriers that the youths consider potential obstacles for usage. Forgetfulness and distraction were identified as a general challenge to beware of. The necessity of visual reminders about the intervention was highlighted and that digital resources could pose a distraction. One third of the participants cautioned that a tendency to ignore, avoid and postpone tedious or difficult tasks might be a barrier for use.

It was generally recommended to avoid overwhelming formats, functions, and content. Receiving massive amounts of information in a passive way, specifically through text, was regarded a huge barrier. One youth said: “*An app with many pages containing symptoms, I dońt think that would be very appealing” (T13).* Another stated that: *“Not a lot of people want a pamphlet from BUP (Unit for child and adolescent psychiatry) with how to handle your ADHD. Many words, then it´s like: ´No! Dad, take it, I dońt even want to see it!´ […] Because it doesńt suit us, that pamphlet from BUP” (T14)*.

The participants regarded negativity and lecturing a potential barrier for engagement. Lastly a lack of variation was discussed as something that might cause the participants to lose interest in the intervention. One youth said: “*Perhaps especially that it (the intervention) is varied, because I think many might get a bit tired and loose concentration and motivation if it´s the same all the time” (T08)*.

### 3.3 Theme 2—Managing ADHD

The theme conveys what areas the youths need help with to manage their ADHD. Sub-themes in this theme are Planning and focus, Regulation and balance, and Social interactions.

#### 3.3.1 Planning and focus

This sub-theme is about challenges related to planning, organizing, and staying focused. A common theme was wanting help to manage planning and organizing. Balancing work and breaks, planning realistically and having useful planning tools in the intervention was mentioned. A need for overview and reminders was discussed, and reminders was considered an essential tool for remembering and keeping track of the day. Challenges with time management was reported by some participants. One participant said: “*I struggle to plan time and such. Being on time to different things. I think that can be smart to focus on because I take things as they come, I don't plan far ahead in advance, I think that I'll fix it when it comes” (T06)*.

The youths wanted tools to promote focus and concentration. The challenge with concentration was stated as an especially prominent issue in everyday school life. One youth said: “*I struggle to concentrate on homework a lot. So, maybe it (the intervention) helps me practice on that. So that I can actually use it when I do my homework. That you can bring in things you need to do and concentrate on into the exercises (in the intervention)” (T05)*.

#### 3.3.2 Regulation and balance

This sub-theme captures a need for learning to balance and regulate emotions, impulses, energies, and thoughts. An important need was being able to control impulses and evaluate consequences. Another major topic they wanted help with was regulating and managing emotions, which was stated to be prominent in people with ADHD. One participant suggested: “*Maybe finding ways to handle feelings. I don't know, many with ADHD has very big emotions, so perhaps learning how to handle that in a healthy way” (T13)*.

A reoccurring topic was the need to find balance and acceptable outlets for big emotions and energy. Physical activity was mentioned as a way of getting an outlet for energy and it was suggested addressing thoughts and feelings by writing it down or talking it through it with someone. One participant stated: “*It´s important to be physically active and not just sit still all the time. You are supposed to use your energy, and not just with words” (T12).* Lastly, a some youths reported that they wanted help managing challenges related to overthinking and chaotic thoughts.

#### 3.3.3 Social interactions

This sub-theme reflects the importance of social interactions and how that might be relevant to address in the intervention. Adapting and being considerate to others was important to the majority of the youths. Learning socially acceptable ways of behaving with others was deemed important and learning social codes and communication was thought to be useful. One youth said: “*You should be able to behave “appropriately” […] I have said stupid things many times without realizing it, and then it turns into dumb situations. So, learning what´s okay and what´s not okay to say” (T03).* Socializing and making friends was a commonly reported challenge.

There were conflicting reports about the relevance of practicing social situations in the intervention. Half of the participants stated it as less relevant [for reasons such as: not believing it reflects reality; feels realistic; you could just do it in real life; and some said they possessed adequate social skills] while the other half reported that testing social situations could be useful. Lastly, half of the youths considered it important to set boundaries for oneself in relation to others, as well as respecting other people's boundaries.

### 3.4 Theme 3—Me and my ADHD

This theme focus on the importance of understanding and embracing the ADHD diagnosis. This includes both the individual with the diagnosis and the people who surround them. The theme consists of two sub-themes: Insight and understanding and Accept and normalization.

#### 3.4.1 Insight and understanding

This sub-theme promotes the importance of insight into the diagnosis in general and individual differences in how this manifest. Information was wanted for the youths themselves and for others to increase their own understanding and to promote understanding from others. One participant pointed out the need for: “*Understanding why you are the way you are and why you are supposed to take medications for example or why it can help. What might help and what potentially does not help and what one should focus on and what to avoid (…)” (T08)*.

A general understanding about ADHD was considered valuable but also getting insight into individual differences in ADHD, and that it might be difficult to create something that fits everyone. One youth stated that: “*ADHD can come across very different, it has to do with how your brain functions. It has a lot to do with your personality and how your ADHD is, or what you struggle most with. I know someone with ADHD who struggle a lot more socially than me” (T14)*.

Information about medications and side-effects was also considered valuable to reassure people who might have a negative view on medications, and to promote understanding about the pros and cons with medications. One third of the participants revealed that their primary source of information about ADHD was from TikTok.

#### 3.4.2 Accept and normalization

The sub-theme reflects experiences with and resistance to prejudice and a want for ADHD to be accepted, normalized, and appreciated. The need to recognize oneself in others was put forward, and perceived to promote positive feelings. One participant mentioned it would be nice to have famous role models with ADHD so people would get positive associations to the diagnosis, and potentially feel better about themselves.

Many participants talked about experiences with prejudice and negative comments about ADHD. One youth shared that: “*If someone knows that you have ADHD then they might back off a bit because they heard that people with ADHD are a bit more crazy than others for instance. It´s been like that for me. A lot of people try to stay away” (T10).* Experiences with negativity directed towards ADHD had promoted a want for the diagnosis to be normalized.

The participants linked ADHD to positive resources like being creative, empathic, fun, energetic and spontaneous, and having guts, and the ability to hyperfocus was often reported to be an advantage. One youth said: “*There's nothing wrong about my ADHD really, but I'm sure some that have it don't understand how good it is to have it” (T16)*.

### 3.5 Theme 4—Balance between support and independence

This theme captures the youths need for balanced support that promote autonomy. The participants stressed the importance of support through positivity and encouragement. One youth said: “*If you master something then you like to get praise. That´s not something you always get”(T10)*.

Having easy access to a remote therapist via a digital intervention was considered useful. The youths expressed that having someone to talk to was an important form of support. The significance of involvement and dialogue between the user and the intervention or supporter was highlighted. Having a say, being heard, and understanding the intention with the things you are instructed to do was discussed by the participants. Support from parents and other facilitators was wanted, but with some conditions. It was suggested to give parents access to parts of the content, but with the possibility to choose what they could access. One participant suggested: “*And then… one should be able to limit… in a way, how much they get access to. […] that one can choose for oneself in a way” (T04).* Lastly, having some freedom of choice in the intervention was deemed important to half of the participants.

## 4 Discussion

### 4.1 Main findings

This study explored youths’ wants and needs from a digital mental health intervention for ADHD, and what they considered as potential barriers for internet-delivered treatment. As mentioned earlier adolescents experience different psychopathology than children, including impairments that lie outside the core symptoms (19). It is therefore important to explore and address these reported impairments in the digital intervention.

The key results indicate a need for an ADHD-friendly solution that addresses challenges in the everyday lives of youths with ADHD. The youths requested that the intervention addresses planning and focus; managing and regulating emotions and energy; and social interactions. Furthermore, the youths wanted insight and understanding, as well as accept and normalization for their ADHD. Many participants requested an intervention that balances support while also addressing their individual need for independence.

### 4.2 Tailoring the intervention to youths with ADHD

The participants wanted treatment tailored to youths with ADHD. They requested a treatment that balance fun and usefulness. Games and VR was considered to meet these criteria, and many wanted rewards and visible progress. Research indicates that “Serious games” designed to promote academic, socio-emotional or cognitive training can facilitate skills related to self-management, planning, and organization (31). Games might be especially rewarding for individuals with ADHD as most games incentivize reaching the next level, and because youth with ADHD are susceptible to the immediacy of feedback as well as the stimuli and multi-modality that games provide (32). Applying rewards aligns with guidelines for developing technological interventions for young people with ADHD (33), and are amongst common elements of evidence-based psychosocial treatments for the user-group (34). Adding gamification or rewards to the digital intervention might enhance treatment motivation and engagement.

Receiving massive and passive information through text was considered an unfit format to convey information. Some related this barrier to their experience of overwhelm and resistance when they received psychoeducation pamphlets when diagnosed with ADHD. It is essential to consider the overlap between reading and oral language challenges and ADHD when developing interventions (35) as previous research (36) has shown that reading difficulties constitute a significant motivational challenge for this user-group. Research also shows that children and youth prefer features such as limited text, videos, the ability to individualize, opportunities to connect with others, and receiving reminders via text message in digital mental health interventions (37). This seems to be in line with the perspectives shared by our participants.

### 4.3 Managing ADHD—tools and strategies for everyday use

Overall, the participants wanted the intervention to provide concrete tools and strategies that they could apply in their everyday lives focusing on three main areas. The first was Planning and Focus, a challenge associated with school life, which many considered their primary challenge. The participants wanted tools for concentration, time-management and learning to plan for a balanced approach to work and breaks. In line with our findings, previous research report that school related challenges are an aspect youths want the ADHD treatment to address (38). More specifically, one study (39) found that young people with ADHD and learning disabilities applied strategies for productivity and time-management such as activity breaks, switching activities, using environmental cues and creating low level stress to overcome academic obstacles. One example was the pomodoro technique for managing and splitting up tasks (39). Our findings and previous research taken together indicate the relevance of delivering practical tools applicable in school settings.

The second area of interest, Regulation and Balance, sprung from the youths’ statements about wanting to manage strong and shifting emotions, impulses, and energy. Many found it difficult to balance energy, getting outlets and requested help approaching energy-demanding tasks. Research supports our findings regarding youths having excess energy and experiences of changing energy levels (38, 40), yet there is little research on concrete approaches to deal with energy-demanding tasks. Strategies for managing energy should be considered in the novel intervention.

The third area of interest was Social interactions. Some of our participants expressed they had no trouble in social interactions while others found it difficult to socialise and make new friends. Previous research shows that youth with ADHD report having peer problems, feel inadequate to their peers and find it hard to establish and sustain friendships (41, 42). A couple of our participants stated clearly that they did not know how to act in social interactions. However, challenges with social interactions in young people with ADHD is not necessarily due to lacking social skills, but rather reflects practical appliance of social skills (43). For example, our participants explained a discrepancy between what they thought they should do, and what they tended to do in situations where they were triggered or felt judged. In these situations, the youths found it difficult to consider other perspectives, and often spoke or acted before thinking. Consequently, evidence-based treatments should address difficulties in social interactions in youth with ADHD, focusing not only on skills but also on emotional dysregulation (44, 45). Learning to manage emotions in the moment and after arousal in social settings is suggested to be an important addition to interventions targeting social functioning in youths with ADHD (44).

### 4.4 Me and my ADHD

The participants wanted insight and understanding regarding their ADHD, and stressed the importance of embracing the diagnosis, finding and receiving acceptance and normalization. The participants shared experiences of judgement and inaccurate stigma associated with their diagnosis or related behaviour, and being dismissed when explaining their challenges. In accordance with our findings, previous research shows that many youths with ADHD feel different from others and report that they need acceptance (41). Addressing and combating stigma related to ADHD is important to facilitate successful ADHD management (46). One way to counteract stigma is by conveying accurate information about mental health disorders such as ADHD, which also enhances self-management and the ability to explain the diagnosis to peers (33, 46).

In general, our participants wanted ADHD to be viewed through a more positive, and less deficit-oriented lens. A study focusing on positive experiences with ADHD (47), found that characteristics such as hyperactivity, unconventional thinking, pursuing new experiences, resilience and growth were perceived as positive sides of ADHD that are useful in certain contexts. There is increasing focus on promoting strength-based perspectives in working with young people with ADHD (48, 49). A recent practitioner review (24) recommended incorporating a focus on strengths and acceptance in treatment to accommodate the unmet needs reported by people with ADHD. Finding ways of promoting understanding, acceptance, normalization, and strengths should be prioritized in development of the novel digital intervention.

### 4.5 Balance between support and independence

Our participants wanted an intervention that balance support and independence. In this study, having contact with a digital therapist for support was considered useful. Human support in digital interventions enable the possibility to contact a person if needed, a therapeutic alliance, and enhanced feeling of accountability (50). The youth wanted the therapeutic climate to promote participation, having choices and dialogue, being informed, and involved in decisions.

Involving youth in decisions regarding their treatment is suggested to promote engagement, participation, satisfaction, outcomes, and cooperation and reduce attitudinal barriers (51, 52). The maturational shift from childhood to youth induce a transgression from passive recipients to active collaborators in decisions regarding their ADHD (41). This aligns with what our participants expressed regarding the need to be involved and have a say in their treatment.

In this study it was suggested that parents could give some support in the intervention, provided that the youths had the possibility to choose what their parents could get access to. The relevance of involving parent-support in the intervention correspond with the above-average parenting needs of children and youths with ADHD (4). Guidelines suggest that digital interventions should facilitate support and encouragement from significant others as this can promote self-efficacy (33). The balance between support and independence is crucial for delivering useful digital treatment that promotes autonomy, and thereby hold the potential to engage and motivate youth.

### 4.6 Core guidelines for developing the digital intervention

Overall, our findings suggest some core guidelines (25) when developing the novel intervention. Based on our findings and the literature on ADHD we decided that the first core guideline will be empowerment. Empowerment encompass self-efficacy, self-worth, power (53), and believing in one-self, and is associated with increased self-determination and influence (54). Support and empowerment can enable youths to take a more active role in managing their ADHD (41). Therapist-support will be one strategy to improve engagement and empowerment. By making youths the primary receiver of the intervention and delivering direct contact with a therapist we want to enhance autonomy. Incorporating some freedom of choice in the intervention as well as focusing on dialogue and involvement in decisions is recommended to promote empowerment.

The second core guideline will be to develop user-friendly design and formats by avoiding overwhelming content. As our results show, youths with ADHD are inclined towards stimulating and interactive treatment and aversive to overwhelming, passive text. Reaching youths through a digital context they are familiar with will likely promote engagement and motivation. The intervention development needs to take the challenges, such as short attention span, as well as strengths, such as hyper-focus, digital competence, and preferences of youth with ADHD into the design considerations.

The third core guideline is to promote a strength-based perspective on ADHD, in a way that enhance user competence, normalization and acceptance. The youths require participation and involvement in line with needs related to the maturational shift from child to youth (41). Not acknowledging youths experiences and taking them seriously can lead to conflict and non-compliance (41).

### 4.7 Limitations

The main limitation in the present study is the lack of sample representativeness given the fact that none of the participants had an immigrant background. The exclusion criteria regarding Norwegian language proficiency might have come across as intimidating to some youths. Using social media channels as a recruitment strategy may have caused distortions. However, the strategy was applied in an attempt to provide a broader recruitment. Due to a lack of adequate participants, social media channels were regarded as a necessary and recommended strategy in order to gain enough participants. Moreover, the different recruitment strategies increase the chance of recruiting a heterogeneous, composed sample of youth with ADHD, thereby reducing the chance of distortions in the sample. Another limitation was that the ADHD diagnosis was not confirmed by a therapist. Further this study has not consulted parents of youth with ADHD. We acknowledge the importance and relevance of family insights and perspectives when tailoring treatment for young people with ADHD, as parents’ perspectives can differ from the youths’ perspectives and contribute to additional understanding. In this study we chose to focus on the youth perspective, as there is little research on their perspective alone. Future studies should include parent perspectives on digital treatment. There are different ways of performing qualitative analysis, and some might prefer more structured analytic practices in line with small q frameworks, such as inter-coder agreements and codebooks (30) to convey some sort of reliability. Small q frameworks might be considered more standardized and easier to replicate by some. We however value the subjective, organic, broad knowledge development provided by the Big Q framework in the reflexive thematic approach and find that the flexibility of the approach has provided us with an opportunity to engage in creative exploring of the perspectives of the youths. We have strived to conduct and report this study in line with Braun and Clarke's (29, 30) steps and values and believe we have done so in a thorough, honest, and transparent manner.

### 4.8 Implications

This study adds to the literature by integrating user-perspectives in the development of digital health interventions. In addition, it contributes to the qualitative research on youths with ADHD, which there are few studies on. As Norwegian youth have high digital competence, we believe that gaining their perspectives on what digital aspects they find appealing or annoying is of great value in the pre-development phase of digital intervention development. We also acknowledge the importance of voicing youth perspectives as they have first-hand experience of the impairments that lie outside the core symptoms of ADHD. The demonstration of the person-based approach in this article could be helpful for others working on developing digital mental health interventions. Future studies should be conducted in order to investigate the feasibility of digital mental health interventions applying person-based approach.

## 5 Conclusion

The participants wanted an intervention that is tailored to their needs as youths with an ADHD diagnosis. The youths wanted strategies for everyday challenges related to Planning and focus; Regulation and balance; and Social Interactions. Furthermore, the youths stressed the need for embracing the diagnosis, and promoting acceptance and normalization. Lastly, the youths wanted an intervention that balances support and independence. This study indicates that youth with ADHD are inclined towards stimulating and inter-active treatment, and aversive to overwhelming, passive content. Therapist supported treatment with empowerment as a core guiding principle is recommended. Varied formats and some freedom of choice should be promoted. In sum, this study provides valuable insights for future intervention development of non-pharmacological treatment for youths with ADHD.

## Data Availability

The datasets presented in this article are not readily available because data generated, analysed, and reported during the current study are not publicly available due to it being potentially identifying, but are available in a slightly shortened, de-identified form from the corresponding author on reasonable request. Requests to access the datasets should be directed to Smiti Kahlon, smiti.kahlon@helse-bergen.no.
