# Supplementary material for: Development of a digital mental health intervention for youth with ADHD: exploring youth perspectives on wants, needs, and barriers
Source: Front Digit Health. 2024 Jul 10;6:1386892. doi: 10.3389/fdgth.2024.1386892 (PMC11266159; doi:10.3389/fdgth.2024.1386892)
Supplement: Supplementary file 1 [file Datasheet1.pdf]

INTERVIEW GUIDE

Information

My name is Maren/Ingeborg/Karin and I work at the Young ADHD-project. Great that you could participate in this interview. I thought we could start by me telling a bit about this study, and then we can do some practical matters before we begin the interview.

In the Young ADHD-project, we aim to develop a digital coping program for adolescents with ADHD. We know that adolescents with ADHD may possess various strengths such as abundant energy, creativity and curiosity. However, we are also aware that ADHD can pose challenges related to concentrating in school, sitting still for extended periods, remembering appointments or managing emotions when faced with unfair situations.

We are aware that there are various ways to cope with and practice challenges associated with ADHD, and we believe that a digital coping program for ADHD could be a valuable tool. That's why we have invited you, along with other adolescents with ADHD, to interviews, as you are the experts on what it's like to have ADHD and how it is to be an adolescence.

This interview will take approximately 30-40 minutes, and there are no 'right' or 'wrong' answers. The questions revolve around your experiences or thoughts on using the internet, apps, or other technology to cope with ADHD, and what might be helpful to include in a digital coping program.

As mentioned in the information sheet, we will be recording the interview to ensure that we capture everything accurately. We take responsibility for securely storing the information you share with us, and your name will not be associated with this interview. Do you have any questions before we begin?

Map the user's knowledge and experiences with digital treatment:

1. **Have you heard of or seen any of these digital technologies before?** (Show images):

VR	**YES NO**
AR	**YES NO**
Game on mobile/tablet	**YES NO**

2. **Have you ever tried apps or other technology to get help with ADHD or used other helping-apps?** **YES NO**

- **Have not tried digital treatment**

Have you heard anything about digital treatment? **YES NO**
If YES: What have you heard?

- **Tried digital treatment**

How do you think it was?
What was positive/negative (the adjective the participant uses)?
Would you like to tell a bit more about it?

Map the user's thoughts and needs related to digital treatment:

3. I will now read out some examples of how digital treatment for ADHD might look, and I ask that you provide feedback on what you think about these examples.

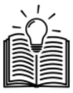

An app where adolescent can learn about ADHD.

This sounds: ____

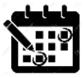

An app to plan your week and get reminders/alerts

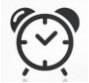

This sounds: ____

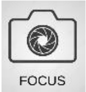

An app for concentration exercises

This sounds: ____

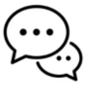

An app where adolescent can chat with the therapist

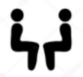

This sounds: ____

An app where the adolescent in a virtual reality (VR-glasses) can e.g. test out different situations such as during school hours, or when they are with friends.

This sounds: ____

A computer game aimed at ADHD where adolescents go through different levels

This sounds: ____

4. a.) What should we focus on in the digital coping program?

b.) We know adolescents with ADHD are different and can struggle with various things. Therefore, we would like to hear your thoughts about these themes (show p. 4). Is there anything special here that might be important for adolescents with ADHD?

Social____

Emotions____

Planning, organization and
focus____

Assessments, control, setting boundaries
____

Medicine____

Coping and self-confidence____

Knowledge about ADHD______________________________________

Energy(level)____

Other?____

c.) Mark the themes that could be useful for adolescents with ADHD

Social

Relations to others
Friends Family
Girlfriend/Boyfriend
School relations
Collaborations Strangers
Gaming

Emotions

Anger Excitement
Boredom Eagerness
Sadness Loneliness
Patience Worry

Planning, organization and focus

Concentration Planning
Organization Keep appointments
Come in time Problem solving

Medicine

Medicine use
Information
Reminders

Assessments, control, setting boundaries

Impulse control Risk assessment
Drug use Setting boundaries
Temptations Curiosity
Consequences

Coping and self-confidence

Coping Self-confidence
Self-esteem

Knowledge about ADHD

Learn about ADHD
How to talk about your own ADHD with others

Other

Everyday situations
Physical activity/exercise
Eating Sleep
Energy level

You may have heard of the comedian Herman Flesvig? From the show «Førstegangstjenesten»? He also has ADHD, and he says that the diagnosis can be an advantage.

What do you think are important advantages or strengths of ADHD?

Mapping the users' interest/thoughts about a digital coping program

6. In what ways do you think digital treatment can make daily life easier for adolescents with ADHD?

7. In what ways do you think it might be challenging for adolescents with ADHD to use a digital program?

8. How do you think a digital treatment can contribute to increasing enthusiasm/motivation for treatment?

9. What do you think the coping program for adolescents with ADHD should not include?

10. What else could be helpful when creating a digital coping program for adolescents with ADHD?

11. Finally; Is there anything we forgot to ask you about? Anything you would like to add or ask about?

- Thank you very much for your valuable inputs. This will greatly assist us in further development!
